# Research on DUAL-ADGAN Model for Anomaly Detection Method in Time-Series Data

**DOI:** 10.1155/2022/8753323

**Published:** 2022-10-26

**Authors:** Xingyu Gong, Xin Wang, Na Li

**Affiliations:** College of Computer Science and Technology, Xi'an University of Science and Technology, Xi'an 710699, China

## Abstract

In recent years, anomaly detection techniques in time-series data have been widely used in manufacturing, cybersecurity, and other fields. Meanwhile, various anomaly detection models based on generative adversarial networks (GAN) are gradually used in time-series anomaly detection tasks. However, there are problems of unstable generator training, missed detection of anomalous data, and inconsistency between the discriminator's discriminant and the anomaly detection target in GAN networks. Aiming at the above problems, the paper proposes a DUAL-ADGAN (Dual Anomaly Detection Generative Adversarial Networks) model for the detection of anomalous data in time series. First, the Wasserstein distance satisfying the Lipschitz constraint is used as the loss function of the data reconstruction module, which improves the stability of the traditional GAN network training. Second, by adding a data prediction module to the DUAL-ADGAN model, the distinction between abnormal and normal samples is increased, and the rate of missing abnormal data in the model is reduced. Third, by introducing the Fence-GAN loss function, the discriminator is aligned with the anomaly detection target, which effectively reduces the anomaly data false detection rate of the DUAL-ADGAN model. Finally, anomaly scores derived from the DUAL-ADGAN model are compared with dynamic thresholds to detect anomalies. The experimental results show that the average F1 of the DUAL-ADGAN model is 0.881, which is better than the other nine baseline models. The conclusions demonstrate that the DUAL-ADGAN model proposed in the paper is more stable in training while effectively solving the problems of anomaly miss detection and discriminator inconsistency with the anomaly detection target in the anomaly detection task.

## 1. Introduction

With the development of artificial intelligence, machine learning and deep learning have been widely used in various fields, such as object tracking [1], visual tracking [2], superresolution reconstruction [3], traffic sign image recognition [4], time-series anomaly detection, and other fields. Time-series anomaly detection is the detection of time-series data points or data segments in a specific scenario where the state of the system is significantly different from the previous normal state [5]. At present, intelligent sensor monitoring equipment is deployed widely in factories, coal mining sites, and other application sites, effectively making up for the problem that manual monitoring cannot detect system failures on time. These monitoring systems record system status information in the form of time-series data by continuously monitoring dynamic changes in the work environment. These time-series data are studied to obtain potential information reflecting the status of the system to enable action to be taken to eliminate potential safety hazards before anomalies cause major accidents. The time-series anomaly detection model is used in practical applications to closely monitor sensor monitoring data patterns, detect anomalies early that could cause a huge disaster, and ensure the safety of the system. Therefore, it is of high theoretical significance and practical value to study the anomaly detection model in time-series data.

Anomaly detection methods based on traditional probability statistics require statistical assumptions about the model through prior knowledge, and the data not conforming to the prior knowledge assumptions are considered as anomalies. However, with the increasing amount of time-series data, these traditional anomaly detection methods, which require extensive domain a priori knowledge, have difficulty handling dynamic and complex time-series data. Therefore, researchers have started to use machine learning methods to detect anomalies. Supervised machine learning-based methods require large amounts of normal data and reliable labeled anomalous data to learn classification models. However, in practical applications, the time-series data are collected from the real world. There are problems of lack of label information, labeling difficulties, and data imbalance. If a supervised anomaly detection method is used, the problem of insufficient anomaly samples in the training samples needs to be addressed. Gao et al. [6–8] used numerical simulation or finite element simulation to simulate fault samples in a mechanical fault detection problem and combined with generative adversarial networks to synthesize fault samples to provide sufficient fault samples for training supervised models. However, when it is difficult to obtain anomaly samples, the unsupervised anomaly detection model is usually used for anomaly detection in time-series data [9]. Most of the existing unsupervised anomaly detection methods partition the time series into subsequences of a certain length and use a clustering model-based approach to detect anomalous values. However, these methods cannot handle potential nonlinear relationships in the time series and lack the ability to capture contextual anomalies. Another class of methods uses the prediction model [10] to predict the system state values and calculate the residuals with the actual values. Residual results above a threshold are considered as anomalies. However, most systems are highly dynamic, and it is difficult to define a range of data that is normal at each moment.

With the development of generative adversarial networks in recent years, this kind of generative deep learning model by adversarial training method has received more and more attention from researchers. GAN [11] models are used to learn high-dimensional complex data distributions in the real world, and they have made great progress in image processing tasks such as medical image synthesis [12] and superresolution reconstruction. Meanwhile, the researchers found that mapping the data inverse to the random potential space and reconstructing the error according to the generator can be effective for anomaly detection tasks. Therefore, GAN has also been extensively studied in the field of image anomaly detection [13, 14]. However, the above studies are all applications of GAN networks in the fields of image generation and image anomaly detection. To study the generative adversarial network model applicable to time-series anomaly detection, an anomaly detection model with a dual generative adversarial network structure is proposed in this paper. The main contributions are as follows:

1. To address the problem of generator training instability in generating sample data for generative adversarial networks, the training stability of GAN networks is improved by introducing the Wasserstein distance satisfying the 1-Lipschitz constraint as the loss function of generative adversarial networks.
2. To address the problem that the generative adversarial network can reconstruct part of the anomalous data when reconstructing the data, which leads the anomaly detection model to miss the anomalous data. The paper designs a time-series prediction module in the generative adversarial network and adds prediction loss to increase the distinction between the anomalous data and the normal data in the process of reconstructing the data in the model so as to reduce the occurrence of anomaly data miss detection.
3. To address the problem that the optimal discriminators trained by generative adversarial networks are inconsistent with the anomaly detection target, the discriminators for the anomaly detection task are trained by introducing the loss function of Fence-GAN into the anomaly detection model, which solves the anomaly data misdetection problem caused by the inconsistency between the optimal discriminators of GAN networks and the anomaly detection target.

The rest of the paper is organized as follows: Section 2 summarizes the related work of this research.  Section 3 describes the DUAL-ADGAN-based anomaly detection method for generative adversarial networks.  Section 4 conducts experiments and evaluation of the proposed model.  Section 5 summarizes the experimental results of this paper to draw conclusions.

## 2. Related Work

In the field of anomaly detection, the lack of a priori knowledge, the difficulty of obtaining data labels, and the imbalance of data samples have caused great challenges in the research of anomaly detection models, but this has also greatly promoted the research and development of anomaly detection methods, which can be mainly divided into the following three categories, anomaly detection methods based on probability statistics, unsupervised anomaly detection methods based on traditional machine learning, and unsupervised anomaly detection methods based on deep learning.

### 2.1. Anomaly Detection Method Based on Probability Statistics

The anomaly detection method based on probability statistics assumes that the data obeys certain distribution, such as Gaussian distribution, through a priori knowledge. The normal data fall in the high probability interval, abnormal data fall in the low probability interval, and the probability of the data in the model is calculated to determine whether it is abnormal or not. Zheng et al. [15] proposed a technique to detect anomalies based on the synthesis of statistical hypotheses and fuzzy sets, using an affiliation function to fuzzy the set of normal and abnormal and identifying data points that do not fit the probability distribution as time-series anomalies. However, traditional statistical hypothesis detection techniques require expert knowledge to estimate the model a priori and are difficult to handle complex data streams. To address this problem, researchers have started to use machine learning approaches to detect anomalies.

### 2.2. Anomaly Detection Methods Based on Traditional Machine Learning

Based on traditional unsupervised machine learning models that measure the similarity between data by means of distance or density, for example, data with distances or densities that differ significantly from most of the data are considered as anomalies. Breunig et al. [16] proposed the Local Outlier Factor (LOF) algorithm, which determines anomalies by calculating the ratio of the average local density of the *k* nearest neighbors of the data object to the local density of the data object itself. Ripan et al. [17] proposed an unsupervised K-means clustering anomaly detection algorithm to detect anomalies by determining the optimal K-value through the contour method, calculating the distance between different samples, and clustering normal and abnormal samples into two clusters. Chen et al. [18] used a K-means++ clustering algorithm to cluster and detect time-series feature data to obtain anomaly data clusters and performed intersection operation on all anomaly clusters to obtain the final set of anomaly detection objects. Liu et al. [19] proposed the isolated forest algorithm, which constructs an isolated tree by continuously selecting random subsamples from data samples to form an isolated forest and determines whether the sample is an outlier based on the size of its path length in the isolated forest. Santiago-Paz et al. [20] proposed an anomaly detection method based on a class of support vector machines (OC-SVM), which detects anomalies by separating the majority of data from the origin by using multiple kernel functions and projecting the data into a high-dimensional space. However, the above traditional unsupervised machine learning-based anomaly detection models all fail to capture temporal correlation by considering the time dimension information in the time-series anomaly problem. This results in their inability to effectively detect contextual anomalies, leading to problems such as false and missed anomaly detection.

### 2.3. Anomaly Detection Methods Based on Deep Learning

Unsupervised anomaly detection methods based on deep learning [21] are popular among researchers because of their ability to deal with complex nonlinear time-dependent problems and their excellent learning capability. The current deep learning-based anomaly detection methods are classified into reconstructed data-based methods and prediction-based methods. The idea of the reconstructed data-based approach is to detect anomalies based on the magnitude of the reconstruction error by reconstructing samples through deep network models such as autoencoder and variational autoencoder. Salehi et al. [22] proposed an autoencoder-based anomaly detection model which improves the robustness of the anomaly detection model by penalizing the unstable codec network layer to force the autoencoder to learn meaningful features. Borghesi et al. [23] proposed an anomaly detection method based on variable autoencoder reconstruction probability, which measures anomalies by probability so that the model does not need a specific threshold to determine anomalies. Feng and Tian [24] proposed to use LSTM-based dynamic state space to capture data dynamics and further improve the accuracy of anomaly detection by Bayesian filtering. However, due to the powerful nonlinear fitting ability of neural networks, the reconstruction-based method can also reconstruct some of the anomalies, leading to anomaly miss detection. The idea of the prediction-based approach is to learn the data distribution of normal behavior by training a prediction model, then predict the data of the next timestamp based on the existing data, and determine the anomaly based on the error between the predicted value and the true value. For example, Hundman et al. [25] demonstrated the effectiveness of employing long short-term memory neural networks in the spacecraft time-series data anomaly detection problem. Wu et al. [10] proposed a stacked LSTM anomaly detection model that uses its powerful learning capability to handle the long and short-term dependence of time-series data and detects anomalous values by the prediction error of the model. However, due to the highly dynamic nature of most systems, it is difficult to define the range of normal data at each moment using a prediction-based anomaly detection approach.

The above-mentioned anomaly detection methods also have problems such as strong model generalization and easy overfitting resulting in anomaly miss detection. The latest work on anomaly detection mainly focuses on anomaly detection models built on the GAN network framework [26]. GAN-based anomaly detection methods were first applied to the image domain. Li et al. [27] proposed a depth translation-based change detection network (DTCDN) for optical and SAR images, using generative adversarial networks to transform the domains of anomalous remote sensing images to achieve remote sensing image change difference detection. Niu et al. [28] constructed defect direction vectors in the potential space of GAN networks to control the defect intensity based on the feature continuity between defects and nondefects in industrial images to achieve defect detection. Most of the anomaly detection methods based on generative adversarial networks detect anomalies by inverse mapping the samples to be tested back to the latent space and reconstructing the samples using generators, while there are also methods that use discriminators and generators together to detect anomalies. Therefore, such methods can also be used to detect anomalies in time-series anomaly detection.

To use GAN for anomaly detection in time series, Li et al. [29] proposed to use the original GAN model to capture the distribution of multivariate time series using discriminators and generators to detect anomalies. However, adopting the original generative adversarial network is training unstable and prone to pattern collapse during the training process. To solve this problem, Geiger et al. [30] combined the Wasserstein distance [31] and the periodic consistency loss to train the generative adversarial network, which enabled the model to effectively reconstruct the time series, and the method addressed the training instability to some extent. However, such deep neural network reconstruction-based anomaly detection models also reconstruct some of the anomalies when reconstructing the data due to their powerful data distribution learning capability. To alleviate this drawback of reconstruction-based anomaly detection methods, Gong et al. [32] proposed a memory-enhanced autoencoder that obtains the encoding through the encoder and then uses it as a query to retrieve the most relevant memory items for reconstruction. Hou et al. [33], in order to regulate the ability of the model to reconstruct normal and abnormal samples, proposed to regulate the reconstruction ability of the model by changing the granularity of the divisions on the feature map. However, the above approaches all use traditional generative adversarial network discriminators to calculate the discriminative loss. Ngo et al. [34] pointed out that discriminators using traditional generative adversarial networks cannot be directly used as a basis for anomaly detection because their training objectives do not coincide with the anomaly detection task and can lead to discriminators that do not effectively distinguish between normal and anomalous data.

The above literature analysis shows that the current GAN-based time-series anomaly detection method still suffers from unstable model training and easy pattern collapse, as well as the problems of anomaly data leakage and inconsistency between the trained optimal discriminator and the anomaly detection target in the anomaly detection process. However, all of these problems affect the anomaly detection performance of the generative adversarial network-based anomaly detection methods.

## 3. Materials and Methods

### 3.1. DUAL-ADGAN Model Overall Framework

**Figure 1:**
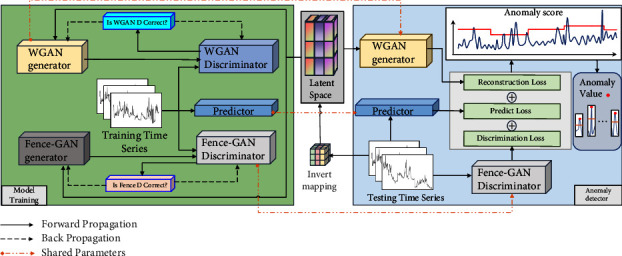
DUAL-ADGAN anomaly detection model.

The core idea of the paper's anomaly detection model is to detect anomalies through three kinds of loss fusion. First, a generator model with the ability to generate normal sample distributions is trained to learn, and when the samples to be tested are input to the generator model, the generator model calculates the reconstruction loss based on the residual difference between the reconstructed samples and the samples to be tested. Second, to increase the differentiation between normal and abnormal samples, the prediction loss is calculated using the residuals between the predictor predictions and the samples to be tested. Third, the discriminative loss is calculated using a discriminator consistent with the goal of the anomaly detection task. Finally, an anomaly score is obtained based on the fused loss values of reconstruction loss, prediction loss, and discriminative loss, which is compared with a dynamic threshold, and data above the dynamic threshold are judged as anomalous. The paper proposes a dual generative adversarial network anomaly detection model structure based on the above ideas, as shown in Figure 1. The anomaly detection model is divided into two networks, the left part is the training network, and the right part is the anomaly detection network. The main task of the training network is to train the generative adversarial network for anomaly detection and the prediction model and then save the parameters of the network models of the completed generators, discriminators, and predictors. In the anomaly detection stage, when the anomaly detection network loads the corresponding model, it can directly call the parameters of the model that has been trained and realize the sharing of parameters from the training network to the anomaly detection network by means of parameter saving and loading. The anomaly detection network maps the samples to be tested to a random potential space, calculates the anomaly scores of the samples to be tested based on the model network parameters shared by the training network, and detects anomalies by comparing them with dynamic thresholds. The red line in the anomaly score block in Figure 1 indicates the dynamic threshold.

The main task of the training network for the DUAL-ADGAN model is to train the parameters of the generator, predictor, and discriminator for anomaly detection network. The generator module can be obtained by training a generative adversarial network with the goal of overlapping the distribution of generated and training data. To prevent GAN training instability or method pattern collapse, this generative adversarial network uses Wasserstein distance as the loss function to improve the reconstructed data capability of the generator. The predictor sets up a time-series task when reconstructing the data and adds the residuals of the predicted data and the data to be measured to the reconstruction loss. In this way, the distinction between normal and abnormal data is increased when reconstructing the abnormal data. The discriminator module is obtained by training a generative adversarial network whose goal is to generate data around the training data, making the discriminator consistent with the goal of the anomaly detection task and thus better able to perform the anomaly detection task.

The anomaly detection network consists of three modules, which are the generator of WGAN, the discriminator of Fence-GAN, and the predictor. These three modules need to share already trained generators of WGAN, discriminators of Fence-GAN, and model parameters of predictors when detecting anomalies. The generator of WGAN is used to calculate the reconstruction loss by inverse mapping the samples to be measured to the random potential space. The noise vector obtained from the random potential space using the generator of WGAN reconstructs the samples and calculates the residuals with the samples to be tested. The discriminator of Fence-GAN is used to calculate the discriminative loss, and the discriminative loss is obtained by directly inputting the samples to be tested into the discriminator of Fence-GAN. The predictor is used to calculate the prediction loss, and the residuals are calculated directly from the predicted values and the samples to be tested. The anomaly score of the sample to be tested is obtained based on the weighted sum of the three loss components of reconstruction loss, prediction loss, and discriminant loss. Data samples that are higher than the threshold value compared with the dynamic threshold value are judged as anomalous.

### 3.2. DUAL-ADGAN Model Training Network

**Figure 2:**
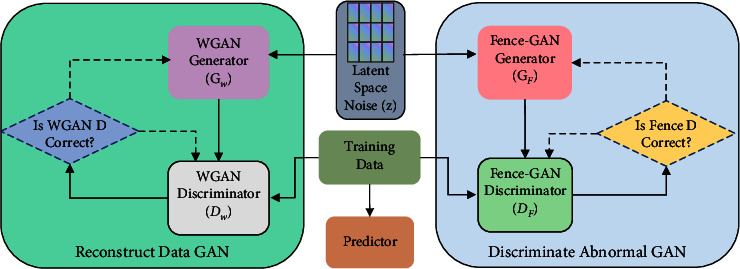
Training network model.

The training network of the DUAL-ADGAN model is mainly divided into three modules, which are data reconstruction module, data prediction module, and anomaly discrimination module; the training network model structure is shown in Figure 2. The main task of the training network module is to train the input data to obtain a generator for reconstructing the data, a predictor for exposing anomalies, and a discriminator model consistent with the anomaly detection task. The neural network model parameters obtained from the training network are shared to the corresponding modules in the anomaly detection network. The input data of the training network are divided into time subseries using a sliding window of size *n* and step size *s*. The time series *X* of length *T* is divided to obtain *m* time subseries, where *m *=* *(*T *−* n*)/*s *+* *1. Based on the experimental results, the sliding window size in this paper is set to 10. In order to fully learn the data distribution when training the model, the step size of the training data is set to 1, and the step size of the test data is consistent with the window size.

#### 3.2.1. Data Reconstruction Module

**Figure 3:**
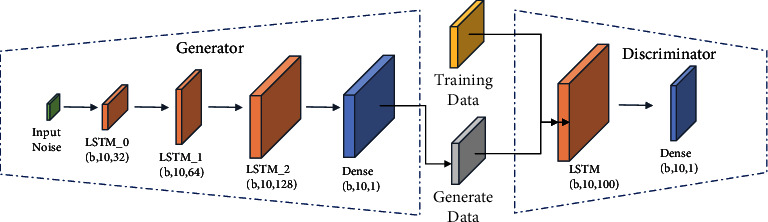
Network structure diagram of the data reconstruction module.

The network structure of the data reconstruction module, shown in the left part of Figure 2, consists of a generator *G*_*W*_ and discriminator *D*_*W*_ based on WGAN. The generator *G*_*W*_ randomly selects the noise vector *z* from the potential space as the input to the generator. The discriminator *D*_*W*_ input is the real training data and the fake data generated by the generator. Through adversarial training, the generator will learn the general distribution of real data and be able to generate realistic fake data to make the discriminator unable to distinguish between real and fake data. In the backpropagation process of adversarial training, the paper uses Wasserstein distance and gradient penalties as the loss functions of the data reconstruction to generate the adversarial network module. The structure of the generator and discriminator network inside the data reconstruction module is shown in Figure 3.

*(1) Generator Construction*

(1) Input layer: the generator obtains the random noise *z*_*W*_ from the random potential space as the input of the generator, the training sample step is 10, and the feature dimension is 1. Therefore, the random noise vector *z*_*W*_ is initialized as shown in equation (1):
  (1) $$\begin{array}{c} z_{W}=Random\left(BatchSize,10,1\right)\text{.} \end{array}$$
(2) Hidden layer: the hidden layer uses a three-layer LSTM network to extract the features of the input vector, and the feature dimensions of each hidden layer are 32, 64, and 128, respectively.
  (2) $$\begin{array}{c} RDG_{h_{t}}^{G}=\text{LSTM}2\left(\text{LSTM}1\left(\text{LSTM}0\left(z_{W},h_{t-1},c_{t}\right)\right)\right)\text{,} \end{array}$$
where RDG is the acronym of the data reconstruction module, *RDG*_*h*_*t*__^*G*^ denotes the hidden layer feature vector extracted by the three-layer LSTM of the data reconstruction module generator, *z*_*W*_ is the random noise vector, *h*_(*t* − 1)_ is the hidden layer state, and *c*_*t*_ is the cellular memory state.
(3) Output layer: the output layer is a layer of fully connected layer whose output dimension is (Batch_Size,10,1) of the generated sample *x*^gen^, and the calculation of *x*^gen^ is shown in

(3) $$\begin{array}{c} x^{gen}=Dense\left(RDG_{h_{t}}^{G}\right)\text{.} \end{array}$$

*(2) Discriminator Construction*

(1) Input layer: the input of the discriminator is the real sample *x* and the generated sample *x*^gen^, which are fed to the discriminator and trained to the discriminator, respectively, and the input sample dimensions are (Batch_Size,10,1).
(2) Hidden layer: the hidden layer uses a layer of LSTM network with a hidden layer feature dimension of 100 and an output dimension of (Batch_Size,10,100).
  (4) $$\begin{array}{c} RDG_{h_{t}}^{D}=LSTM\left(x,x^{gen},h_{t-1},c_{t}\right)\text{,} \end{array}$$
where *RDG*_*h*_*t*__^*D*^ denotes the hidden layer feature vector extracted by the discriminator LSTM network of the data reconstruction module, *x* and *x*^gen^ denote the real and generated samples, respectively, *h*_(*t* − 1)_ is the hidden layer state, and *c*_*t*_ is the cellular memory state.
(3) Output layer: the output layer is a fully connected layer with the discriminant vector *D* of dimension (Batch_Size,10,1).

(5) $$\begin{array}{c} D=Dense\left(RDG_{h_{t}}^{D}\right)\text{,} \end{array}$$

where *RDG*_*h*_*t*__^*D*^ is the hidden layer feature vector extracted by the LSTM network and *D* denotes the discriminant result of the discriminator for the real and generated samples.

*(3) The Loss Function*. The generative adversarial network of the data reconstruction module uses a combination of Wasserstein distance and gradient penalty (GP) as the loss function, and the mathematical expression of the Wasserstein distance is shown in

(6) $$\begin{array}{c} W\left(P_{r},P_{g}\right)=E_{\left(x,x^{gen}\right)∼\gamma }\;{\;}_{\gamma ∼\Pi \left(P_{r},P_{g}\right)}^{inf}\left[\left\|x-x^{gen}\right\|\right]\text{,} \end{array}$$

where *P*_*r*_ and *P*_*g*_ are the true sample distribution and the generated sample distribution, respectively, and ∏(*P*_*r*_, *P*_*g*_) is the set of all possible joint distributions combined by*P*_*r*_ and *P*_*g*_. For each possible joint distribution *γ*, the true sample *x* and the generated sample *x*^gen^ can be sampled from the joint distribution, the distance ‖*x* − *x*^gen^‖ between the pair of samples is calculated, and finally, the lower bound is taken among all possible joint distributions, which is the Wasserstein distance. The loss function of WGAN is the Wasserstein distance from the generating sample distribution to the real sample distribution, and the WGAN loss function is shown in

(7) $$\begin{array}{c} L_{loss}=E_{z∼p_{g}\left(z\right)}\left[D\left(z\right)\right]-E_{x∼p_{r}\left(x\right)}\left[D\left(x\right)\right]\text{,} \end{array}$$

where *𝔼*_*z*∼*p*_*g*_(*z*)_ denotes the expectation of the potential spatial noise vector distribution, *𝔼*_*x*∼*p*_*r*_(*x*)_ denotes the expectation of the real data distribution, and *D* (∗) is the discriminator.

In order to make the norm of *L*_loss_ gradient bounded and satisfy the 1-Lipschitz constraint, a gradient penalty term (GP, gradient penalty) is added to the loss function of WGAN to limit the gradient variation range, and the mathematical expression of the gradient penalty is shown in

(8) $$\begin{array}{c} GP=\lambda E_{\overset{\wedge }{x}∼p\left(\overset{\wedge }{x}\right)}\left[{\left({\left\|\left.\nabla_{\overset{\wedge }{x}}\left(D\right.\left(\overset{\wedge }{x}\right)\right)\right\|}_{2}-1\right)}^{2}\right]\text{,} \end{array}$$

where *λ* is the gradient penalty term coefficient and ‖∇_*x*_(*D*(*x*)‖_2_ is the gradient of the discriminator.

The Wasserstein distance loss *L*_loss_ combined with the gradient penalty term GP is used as the loss function of the data reconstruction module, and the final WGAN-GP discriminator loss function is shown in

(9) $$\begin{array}{c} D_{loss}=E_{z∼p_{g}\left(z\right)}\left[D\left(z\right)\right]-E_{x∼p_{r}\left(x\right)}\left[D\left(x\right)\right]+\lambda E_{\overset{\wedge }{x}∼p\left(\overset{\wedge }{x}\right)}\left[{\left({\left\|\nabla_{\overset{\wedge }{x}}\left(D\left(\overset{\wedge }{x}\right)\right.\right\|}_{2}-1\right)}^{2}\right]\text{.} \end{array}$$

The first of these terms is the discriminator loss function using the Wasserstein distance, and the second is the gradient penalty term.

Finally, the generators and discriminators of the data reconstruction module are trained alternately using the loss function backpropagation loop until the generative adversarial network reaches Nash equilibrium, which completes the training of generators that can be used for data reconstruction.

#### 3.2.2. Data Prediction Module

Reconstruction-based deep network models for anomaly detection, both autoencoder and generative adversarial networks, are also able to reconstruct some of the anomalies when reconstructing the data due to their strong nonlinear fitting ability, which can easily lead to anomaly miss detection. In order to improve the detection performance of anomaly detection model, the paper designs a time-series prediction task. The core idea of this task is to add some tasks that can be easily accomplished for normal data for the anomaly reconstruction phase, while for anomalous data, the time-series prediction task cannot be effectively accomplished because of the missing information of normal data distribution. The normal and abnormal are distinguished according to the completion degree of the prediction task. The data prediction module in the DUAL-ADGAN model is shown as a predictor in the middle part of Figure 2. The data prediction module uses RNN as the prediction model, and the RNN model is trained to learn normal time-series features.

The RNN model training first feeds the current moment training sample *x*_*t*_ and the previous hidden layer RNN unit output *h*_(*t* − 1)_ into the current moment RNN unit and calculates the hidden layer state h_t of the last unit output of the RNN, which is the temporal feature in the time-series sample extracted by the RNN model, and its calculation process is shown in

(10) $$\begin{array}{c} h_{t}=\tanh\;\left(w_{hh}h_{t-1}+w_{hx}x_{t}\right)\text{,} \end{array}$$

where *w*_*hh*_ is the hidden layer trainable parameter matrix, *w*_*hx*_ is the input sample trainable parameter matrix, tanh is the activation function, *h*_(*t* − 1)_ is the previous hidden layer output state, *h*_*t*_ is the hidden layer output at the current moment, and *x*_*t*_ is the input training sample.

The RNN model obtains the final prediction result by linear variation of the hidden layer state, and the variation process is shown in (11), where the input is the training data *x*_*t*_ and the hidden layer state *h*_*t*_ and the output is the prediction value *y*_*t*_.

(11) $$\begin{array}{c} y_{t}=w_{hy}h_{t}\text{,} \end{array}$$

where *w*_*hy*_ is the trainable parameter matrix that makes linear changes to the output, *h*_*t*_ is the hidden layer state, and *y*_*t*_ is the final predicted value.

The data prediction module in the DUAL-ADGAN model predicts the samples to be tested based on the trained RNN model, calculates the mean square error between the predicted value and the true value of the samples to be tested, obtains the prediction loss, and finally adds the prediction loss to the reconstruction loss in order to use the prediction loss to increase the differentiation between normal and abnormal samples in the reconstruction process of the generative adversarial network.

#### 3.2.3. Data Discriminator Module

The structure of the data anomaly discriminator module is shown on the right side of Figure 2 (Discriminate Abnormal GAN). The module consists of a generator *G*_*F*_ and a discriminator *D*_*F*_. The generator *G*_*F*_ obtains the noise vector *z* of dimension (Batch_Size,10, 1) from the random potential space as the input of the generator. The input of the discriminator is divided into the generated data and the real data, and the input sample dimensions are (Batch_Size,10, 1). The training goal of the data discriminator module is to have the generated data distribution around the real data distribution.

**Figure 4:**
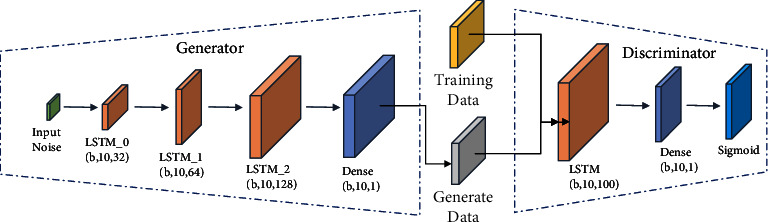
Network structure of the data discriminator module.

The number of network layers and the input and output dimensions of the generator *G*_*F*_ and discriminator *D*_*F*_ in the data discriminator module are the same as those in the data reconstruction module, but the discriminator *D*_*F*_ in the data discriminator module needs to complete the binary classification task, so the sigmoid activation function is added to the last output layer of the discriminator *D*_*F*_. The network structure of the data discriminator module is shown in Figure 4.

The network structure of the data discriminator module is similar to that of the data reconstruction module, with the main difference being the use of different loss functions. The idea of the loss function of the traditional generative adversarial network is to encourage the distribution of the generated samples to overlap with the distribution of the real samples, and the loss function of the traditional generative adversarial network discriminator is shown in

(12) VD maxD,V=Ex∼prxlog Dx+Ez∼pgzlog 1−DGz,

where *𝔼*_*x*∼*p*_*r*_(*x*)_ denotes the expectation of the true sample distribution, *𝔼*_*z*∼*p*_*g*_(*z*)_ denotes the expectation of the noise distribution, *D* (∗) is the discriminator, and *G* (∗) is the generator.

The optimal discriminator *D*^*∗*^(*x*) is obtained by using the loss function in (12), fixing the generator *G*, and deriving it for the discriminator D(x), as shown in

(13) $$\begin{array}{c} D^{*}\left(x\right)=\frac{P_{r}\left(x\right)}{P_{r}\left(x\right)+P_{g}\left(x\right)}\text{,} \end{array}$$

where *D*^*∗*^(*x*) is the optimal discriminator, *P*_*r*_(*x*) is the true sample distribution, and *P*_*g*_(*x*) is the generated data sample distribution.

The loss of traditional generative adversarial networks encourages the distribution of the generated samples to overlap with that of the real samples, which means that *P*_*r*_=*P*_*g*_. After the discriminator converges to the optimal case, the discriminator gets a discriminant probability close to 1/2 when the Nash equilibrium point is reached. The discriminator has no direct relevance to the anomaly detection problem.

Therefore, in order to solve the problem that the discriminator is inconsistent with the target of the anomaly detection task. The goal of the anomaly discriminator module in the paper is to train so that the data distribution generated by the generator surrounds the real data instead of overlapping with the real data distribution. To achieve a generator that generates a data distribution around the real data distribution, the data discrimination module uses the loss function of Fence-GAN with the mathematical expression shown in

(14) $$\begin{array}{c} F\left(G,D\right)=E_{z∼p_{g}\left(z\right)}\left[\log\;\left(\left|\alpha -D\left(\left.G\left(z\right)\right|\right)\right.\right)\right]+\beta *\;\frac{1}{E_{z∼p_{g}\left(z\right)}\left[{\left\|G\left(z\right)-\mu \right\|}_{2}\right]}\text{,} \end{array}$$

where *α* ∈ (0, 1), when the points are generated within the true data distribution, the discriminator will give a score higher than *α*, and thus the generator will be penalized. When the generated data are far from the true data distribution, the score given by the discriminator will be less than *α*, and the generator will be penalized as well. The second term is used to maximize the center distance *μ* between the generated data points and the true data points to encourage the generated points to cover the boundary of the true data distribution. *β* is the hyperparameter of the second loss condition weight, which controls the distance of the generated data sample distribution from the true sample.

Finally, the data discriminator module trains the generator and discriminator alternately according to the loss function of (14) until the sample distribution generated by the generator is around the real sample distribution, which completes the training of the data discriminator module and thus completes the training of the discriminator that can be used to discriminate abnormal data.

The training network calculation process is shown in the DUAL-ADGAN training network model pseudocode Algorithm 1 describes the process of model training.

### 3.3. DUAL-ADGAN Model Anomaly Detection Network

**Figure 5:**
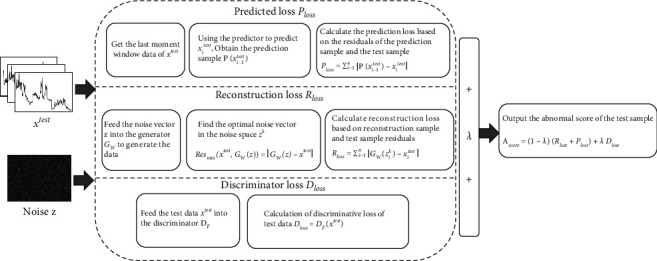
Schematic diagram of the abnormality detection process.

The anomaly detection network mainly calculates three partial loss values and judges the anomalous data together based on the three loss values. The schematic diagram of the abnormality detection process is shown in Figure 5.

The input is the data to be measured *x*^test^ and the noise vector *z* in the potential space. Generator *G*_*W*_ learns the mapping z⟶*x*^test^ in the random potential space and generates the time subsequence *x*^test^ using the noise vector *z* in the random potential space. In order to find the optimal *z*^*k*^ from the random potential space by inverse mapping, it is first necessary to randomly sample *z*^1^ from the potential space, feed it into the generator *G*_*W*_ to the reconstructed sample *G*_*W*_(*z*^1^), and then calculate the residuals of *G*_*W*_(*z*^1^) and the data *x*^test^ to be measured. The noise vector *z* in the potential space is cyclically updated by minimizing the residual Res result to find the optimal noise vector *z*^*k*^. When the preset minimum residual Res_min_ or the maximum cycle is reached, the update is stopped to find the optimal *z*^*k*^. The minimized residual Res_min_ in the paper is 0.1, and the maximum cycle period is 20 times. The mathematical expression of the minimized residuals is shown in

(15) $$\begin{array}{c} Res_{\min}\left(x^{test},G_{W}\left(z\right)\right)=\left|G_{W}\left(z\right)-x^{test}\right|\text{.} \end{array}$$

The optimal *z*^*k*^ found from the potential space is fed into the generator of WGAN to obtain the reconstructed sample *G*_*W*_(*z*^*k*^). The reconstruction loss is calculated by computing the residual between the sample to be measured *x*^test^ and the reconstructed sample *G*_*W*_(*z*^*k*^), as shown in

(16) $$\begin{array}{c} R_{loss}=\overset{n}{\underset{i=1}{\sum }}\left|G_{W}\left(z_{i}^{k}\right)-x_{i}^{test}\right|\text{,} \end{array}$$

where *n* is the time step of the subsequence. *G*_*W*_(*z*_*i*_^*k*^) is the generated data that matches the normal data distribution and is most similar to the test sample by inverse map finding.

The data prediction module in the anomaly detection network calculates the residuals between the predicted values and the samples to be tested to obtain the prediction loss by setting up a time-series prediction task. The prediction loss calculation formula is shown in

(17) $$\begin{array}{c} P_{loss}=\overset{n}{\underset{i=1}{\sum }}\left|P\left(x_{i-1}^{test}\right)-x_{i}^{test}\right|\text{,} \end{array}$$

where *x*_*i*−1_^test^ indicates that the previous subsequence sample is used as the output of the prediction model and the predicted value *P*(*x*_*i*−1_^test^) is used as the residual with the test sample *x*_*i*_^test^ to obtain the prediction loss.

The discriminator of Fence-GAN is used to calculate the discriminative loss, and the discriminative loss *D*_loss_ = *D*_*F*(*x*^test^)_ can be obtained directly by inputting the sample to be tested into the discriminator. The final anomaly score *A*_Score_ is calculated as shown in

(18) $$\begin{array}{c} A_{Score}=\left(1-\lambda \right)\left(R_{loss}+P_{loss}\right)+\lambda \;D_{loss}\text{,} \end{array}$$

where *R*_loss_, *P*_loss_, and *D*_loss_ are reconstruction loss, prediction loss, and discriminant loss, respectively. *λ* is the relative importance of hyperparameter control loss values.

The anomaly score A_*Score*_ is calculated according to (18), using the threshold method to determine whether there is an anomaly in the subsequence. The paper uses a sliding window adaptive technique to determine the threshold value. The threshold value within this window is calculated based on the anomaly score within the sliding window. When the anomaly score of a subsequence is greater than the threshold value in the window where it is located, the subsequence is determined to be an anomaly subsequence. The size of the sliding window *w* determines the number of thresholds for setting the anomaly score, and the size of the step size *s* determines the fineness of the anomaly detection. According to the “3*σ* guidelines,” the threshold in each sliding window is set to the mean *μ* plus 3 times the standard deviation *σ*. The mathematical expression of the dynamic threshold in each window is shown in

(19) $$\begin{array}{c} Threshold=\mu +\left(3*\;\sigma \right)\text{.} \end{array}$$

Finally, the abnormal score *A*_Score_ output from the DUAL-ADGAN model is compared with the dynamic threshold Threshold, and the data *x*^test^ with abnormal score greater than or equal to the dynamic threshold is detected as abnormal data *x*_Anomaly_^test^ and those smaller than the dynamic threshold are normal data *x*_Normal_^test^ whose mathematical expression is shown in

(20) $$\begin{array}{c} x^{test}=\left\{\begin{array}{c} x_{\text{Anomaly}}^{test}\;\left(A_{Score}\geq Threshold\right),\\ x_{\text{Normal}}^{test}\;\left(A_{Score}<Threshold\right). \end{array}\right. \end{array}$$

The anomaly detection network calculation process is shown in the DUAL-ADGAN anomaly detection network model pseudocode Algorithm 2 describes the process of model anomaly detection.

## 4. Results and Discussion

### 4.1. Experimental Setup

#### 4.1.1. Experimental Data

The experiments were conducted using publicly available datasets from the NAB (Numenta Anomaly Benchmark) database for model validation experiments. NAB is a benchmark database for evaluating anomaly detection models and consists of over 50 collections of time-series data with labeled anomalies, each with 1000–22000 data instances, for a total of 365,551 data points. This experiment uses RealAdExchange-CPC, RealTraffic-SPEED, and RealTraffic-TravelTime from NAB as the experimental dataset. The RealAdExchange dataset counts the click through rate data of online ads, which is characterized by the cost per click (CPC). The CPC has 4808 training sets and 1539 test sets. The RealTraffic dataset is collected by the Minnesota Department of Transportation from real-time traffic data in the Twin Cities metropolitan area of Minnesota, including the vehicle travel speed dataset and the total vehicle travel time dataset, with 6000 entries in the SPEED training set and 1128 entries in the test set. The TravelTime training set has 4664 entries, and the test set has 2501 data.

#### 4.1.2. Model Parameters

Network structure parameters: the generators of both WGAN and Fence-GAN consist of a 3-layer LSTM with 100 hidden units. The discriminators of both WGAN and Fence-GAN consist of a one-layer LSTM with 100 hidden units. The discriminators of WGAN do not use an activation function. The predictor uses a single-layer RNN with 50 hidden units, and an activation function is ReLU. The network structure parameters are shown in Table 1.

#### 4.1.3. Evaluation Indicators

The experiments use four standard classification evaluation metrics, accuracy, precision, recall, and F1 value metrics, to measure the performance of the anomaly detection model. Accuracy is the accuracy of the test, which indicates how many abnormal and normal samples are judged to be accurate among all samples. Precision is the precision of the detection, which indicates how many samples of the detected anomalous sequences are genuine anomalies. Recall is the recall rate, which indicates how many samples in the original actual sequence of anomalies were correctly identified. The F1 value is the summed average of precision and recall, taking into account the precision and recall of the classification model. In practice, when both recall and precision reach a certain level and want to continue to improve, you need to face the problem of choice because recall and precision affect each other. In scenarios such as industrial manufacturing and data centers, every anomaly needs to be detected as much as possible, as missed anomalies will have incalculable consequences. Therefore, this paper adopts the F1 value as the main metric to measure the anomaly detection performance.

#### 4.1.4. Baseline Model

The following nine anomaly detection models were used as baseline models to compare with the anomaly detection models proposed in this paper. Four traditional machine learning-based unsupervised anomaly detection models, K-means clustering (K-means), one class of support vector machine (OC-SVM), local anomaly factor (LOF), and isolated forest (IF), were selected as the comparison models, respectively. Five deep learning-based unsupervised anomaly detection models, LSTM-AE, NSIBF, MAD-GAN, Fence-GAN, and Tad-GAN, were also selected as comparison models.

#### 4.1.5. Experimental Protocol

The experiment is divided into three steps, which are as follows:

 
*Step 1*. Compare the anomaly detection performance of DUAL-ADGAN with the other nine unsupervised anomaly detection baseline models on three datasets, RealAdExchange-CPC, RealTraffic-SPEED, and RealTraffic-TravelTime.
 
*Step 2*. The anomaly detection network of DUAL-ADGAN consists of three submodules, which are the generator of WGAN, the discriminator of Fence-GAN, and the predictor. To analyze and demonstrate the role of each submodule of DUAL-ADGAN in anomaly detection, the following three models are set in this part, which are Wad-GAN, WganG_FenceD, and the combined model DUAL-ADGAN with the addition of predictor.
 
*Step 3.* Compare the stability of DUAL-ADGAN anomaly detection models using the original generative adversarial network loss function and Wasserstein distance-based as the loss function, respectively, during training.

### 4.2. Experimental Results

#### 4.2.1. Anomaly Detection Model Performance Comparison Experiment

This section compares the DUAL-ADGAN anomaly detection model proposed in the paper with nine other baseline models on three datasets, RealAdExchange-CPC, RealTraffic-SPEED, and RealTraffic-TravelTime, according to the experimental protocol setup. Four metrics are used in the research to assess the effectiveness of anomaly detection models: accuracy, precision, recall, and F1 value.  Table 2 displays the detection result data from the ten anomaly detection models on the three datasets.

From Table 2, we can see that the DUAL-ADGAN anomaly detection model has the highest accuracy, recall, and F1 values on the three datasets RealAdExchange-CPC, RealTraffic-SPEED, and RealTraffic-TravelTime. Although the accuracy rate decreases, overall, the average F1 and average recall of the DUAL-ADGAN model on the three datasets are 0.881 and 0.931, respectively, which are better than the other comparison models, indicating that the detection model proposed in the paper outperforms the other nine comparison models in terms of anomaly detection.

**Figure 6:**
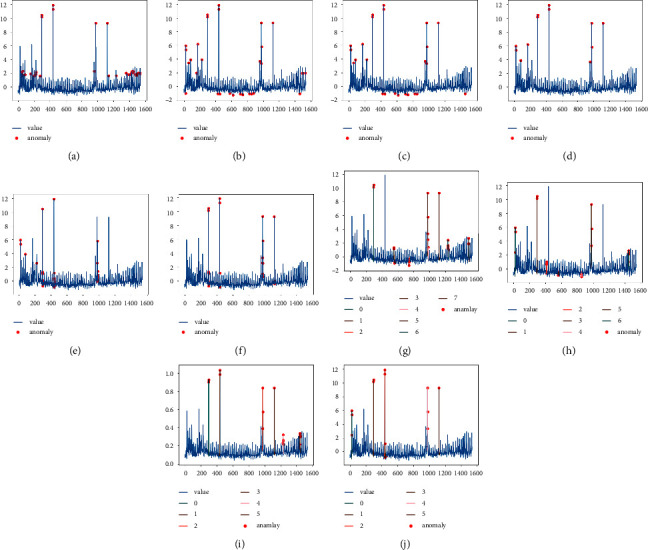
Detection results of the ten anomaly detection models on the RealAdExchange-CPC dataset. (a) K-means. (b) OC-SVM. (c) LOF. (d) IF. (e) LSTM-AE. (f) NSIBF. (g) MAD-GAN. (h) Fence-GAN. (i) Tad-GAN. (j) DUAL-ADGAN.

**Figure 7:**
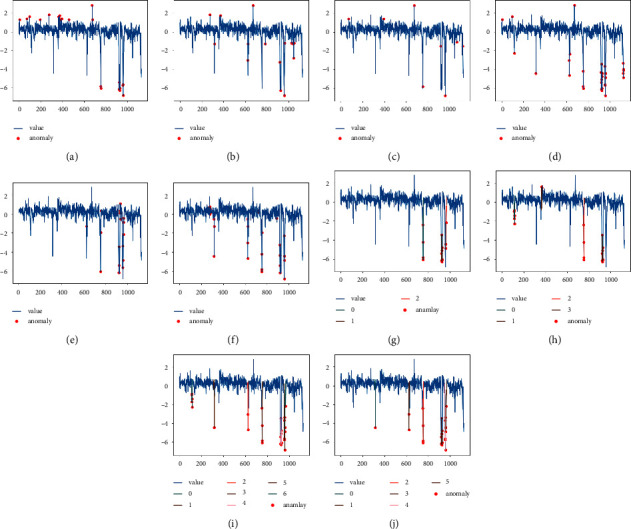
Detection results of the ten anomaly detection models on the RealTraffic-SPEED dataset. (a) K-means. (b) OC-SVM. (c) LOF. (d) IF. (e) LSTM-AE. (f) NSIBF. (g) MAD-GAN. (h) Fence-GAN. (i) Tad-GAN. (j) DUAL-ADGAN.

**Figure 8:**
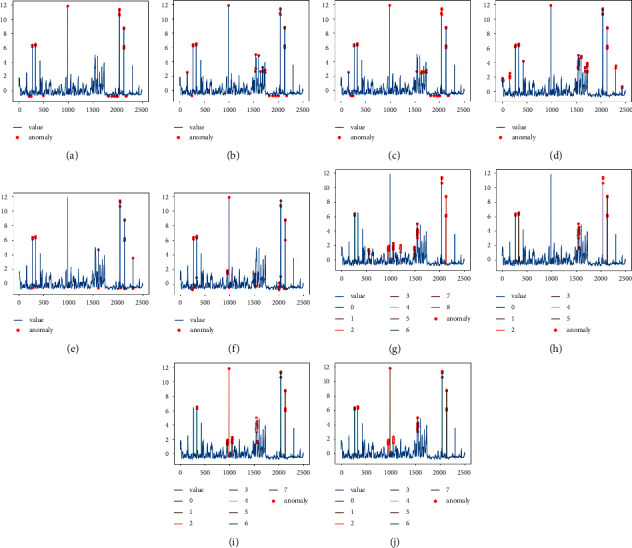
Detection results of the ten anomaly detection models on the RealTraffic-TravelTime dataset. (a) K-means. (b) OC-SVM. (c) LOF. (d) IF. (e) LSTM-AE. (f) NSIBF. (g) MAD-GAN. (h) Fence-GAN. (i) Tad-GAN. (j) DUAL-ADGAN.

Figures 6–8 show the anomaly detection results of the ten models on the three datasets RealAdExchange-CPC, RealTraffic-SPEED, and RealTraffic-TravelTime. The blue curve in the figure is the time-series data containing the anomalies, and the red points are the anomalous values detected by the anomaly detection models. Figures 6(g)–8 are the result plots of the four anomaly detection models based on generative adversarial networks, where the colored lines are the windows of detected anomaly time-series data.

From the three sets of anomaly detection result graphs, it can be seen that the anomaly detection effect based on deep learning models is better than traditional machine learning models in general, and the average F1 value of the six deep learning methods is improved by 13.21% compared to the four traditional machine learning methods. Among them, three models, K-means, OC-SVM, and LOF, have low anomaly detection performance, with average F1 values of 0.568, 0.663, and 0.683 on the three datasets, respectively. The reason for the low F1 values of the above three models is that these models cannot capture contextual correlations, resulting in more false detections. The detection results of these three models are shown in Figures 6–8(c). The detection performance of the isolated forest model is relatively stable, but the model cannot effectively use the nonlinear relationship between features, resulting in a reduced ability of the model to isolate anomalies, and the detection effect of the isolated forest model is shown in Figures 6–8(d). The baseline models based on deep learning include LSTM-AE, NSIBF, MAD-GAN, Fence-GAN, and Tad-GAN as well as the DUAL-ADGAN model proposed in the paper. A comparative analysis of DUAL-ADGAN with these three models on the three datasets shows that the average F1 value of the DUAL-ADGAN model on the three datasets is improved by 15.6%, 11.6%, 13.3%, 6.5%, and 1.5% compared to LSTM-AE, MAD-GAN, Fence-GAN, Tad-GAN, and NSIBF, respectively. The experimental results verify the effectiveness of the DUAL-ADGAN model for the time-series anomaly detection problem.

On the RealAdExchange-CPC dataset, DUAL-ADGAN has the highest accuracy and recall rate, and the accuracy rate decreases compared to NSIBF, but in practice, it is necessary to balance the accuracy and recall rates, so F1 is used as the main evaluation metric, and the F1 value of DUAL-ADGAN is improved by 1.8% compared to NSIBF. The recall rate of Tad-GAN on this dataset is second only to DUAL-ADGAN, but its accuracy rate is lower, mainly because Tad-GAN detects more outliers, but there are a small number of false detections among them.

On the RealTraffic-SPEED dataset, DUAL-ADGAN has the highest accuracy and recall rate, and the accuracy rate is lower than NSIBF and MAD-GAN models, but MAD-GAN has a lower recall rate, mainly because the MAD-GAN model uses the original generative adversarial network loss function, which causes its training process to be unstable and the model does not fully learn to the distribution of normal data. As a result, MAD-GAN only detects a small number of obvious anomalies in this dataset, so its precision rate is high, and the recall rate is low, as shown in Figure 6(g). DUAL-ADGAN achieves the highest recall rate of 0.9 in this dataset, but the precision rate decreases, mainly because its anomaly exposure module exposes a large number of anomalous samples while also treating a small number of normal samples as anomalous, which causes the precision rate. The decrease in the accuracy rate is caused by the fact that its anomaly exposure module exposes a large number of anomalous samples while also treating a small number of normal samples as anomalous.

On the RealTraffic-TravelTime dataset, DUAL-ADGAN only has a slightly lower accuracy rate than NSIBF and Fence-GAN, but the highest accuracy, recall, and F1 values. The main reason is that DUAL-ADGAN uses WGAN generator and Fence-GAN discriminator and predictor together to detect anomalies, which makes the model more stable and accurate in anomaly detection, its recall rate and F1 value are the highest on this dataset, and its detection result graph is shown in Figure 7(j).

Combining the above experimental comparative analysis, the anomaly detection performance of the DUAL-ADGAN anomaly detection model proposed in the paper outperforms other baseline models. This fully demonstrates that using Wasserstein distance as the loss function of the generative adversarial network, adding predictors to expose anomalies, and replacing discriminators that are inconsistent with the anomaly detection target can effectively improve the performance of the time-series anomaly detection model.

#### 4.2.2. DUAL-ADGAN Model Ablation Experiments

According to the experimental protocol setup, this part performs ablation model comparison experiments on DUAL-ADGAN anomaly detection models on three datasets, RealAdExchange-CPC, RealTraffic-SPEED, and RealTraffic-TravelTime. DUAL-ADGAN consists of three submodules, which are the generator module of WGAN, the discriminator module of Fence-GAN, and the predictor module. To analyze and demonstrate the role of each submodule of DUAL-ADGAN in anomaly detection, the following three groups of models are set up in this section: the WGAN anomaly detection model (Wad-GAN), the anomaly detection model combining the WGAN generator module and the Fence-GAN discriminator module (WganG + FenceD), and the combined model DUAL-ADGAN (WganG + FenceD + Predictor) proposed in the paper. The experiments used accuracy, precision, recall, and F1 values to evaluate the anomaly detection performance of each model in the ablation experiments.  Table 3 shows the results of the ablation experiments for the three models on the three data sets.

From the ablation experimental results in Table 3, it can be seen that the WganG_FenceD anomaly detection model has improved the accuracy and recall rate on RealTraffic-SPEED and RealTraffic-TravelTime datasets compared with the Wad-GAN model, and the experimental data indicate that WganG_FenceD has corrected the misdetected anomalies in the Wad_GAN model to some extent and reduced the false detection rate. It is demonstrated that the generative adversarial network discriminator with the training objective of overlapping real and generated samples cannot be directly used for anomaly detection, and the discriminator with generated samples around real samples trained by Fence-GAN can detect anomalous samples by the distribution of the samples to be tested. The experimental results show that the discriminator of Fence-GAN combined with the generator of WGAN can solve the problem that the discriminator of the original generative adversarial network is inconsistent with the anomaly detection target.

The combined model DUAL-ADGAN with the data prediction module added to the WganG_FenceD anomaly detection model has a significant improvement in accuracy, recall, and F1 values compared to the Wad-GAN and WganG_FenceD anomaly detection models but has a small decrease in precision in CPC and TravelTime datasets. The main reason is due to the fact that the data prediction module in the DUAL-ADGAN model also exposes a small number of indistinguishable normal values as anomalies when they are exposed, but overall the model has a more significant improvement in recall. Through the above analysis, the addition of the data prediction module can improve the overall performance of the model and verify the effectiveness of the anomaly exposure mechanism.

In the CPC dataset, Wad-GAN has the highest precision rate, but the accuracy, recall, and F1 values are lower compared to the other two models. It is mainly because the Wad-GAN model uses the generator and discriminator of the traditional generative adversarial network to do anomaly detection, and its discriminator is not consistent with the target of anomaly detection, so Wad-GAN mainly judges the anomaly through the generator reconstruction loss, so the model only detects the anomaly with more obvious reconstruction error, which causes the problem of high precision of detection results and low recall rate. In the SPEED dataset, the performance of all three models decreases due to the more complex trend of the data, but the DUAL-ADGAN model detects anomalies jointly by the three-part loss, which makes the model more robust, so its accuracy, precision, recall, and F1 value are the highest in the SPEED dataset. In the TravelTime dataset, the WganG_FenceD model has the highest precision rate, but the difference with the precision rate of DUAL-ADGAN is small; meanwhile, the recall rate and F1 of the WganG_FenceD model are lower than those of the DUAL-ADGAN model, which proves that the addition of predictor module can effectively solve the problem of anomaly miss detection.

#### 4.2.3. Comparison of Model Training Stability

The anomaly detection model DUAL-ADGAN that uses the original generative adversarial network loss function to train the model will have training difficulties and pattern collapse; in order to improve the stability of model training, the DUAL-ADGAN model adopts the Wasserstein distance as the loss function of the reconstructed data generative adversarial network. To verify the stability of the model training, this part of the experiment compares the stability of the DUAL-ADGAN anomaly detection model using the original generative adversarial network loss function and the Wasserstein distance-based loss function during training. The experiments use three datasets, RealAdExchange-CPC, RealTraffic-SPEED, and RealTraffic-TravelTime, to train the models. The data window in the model training is divided into 10, the step size is 1, and the training epoch is 400 rounds.

**Figure 9:**
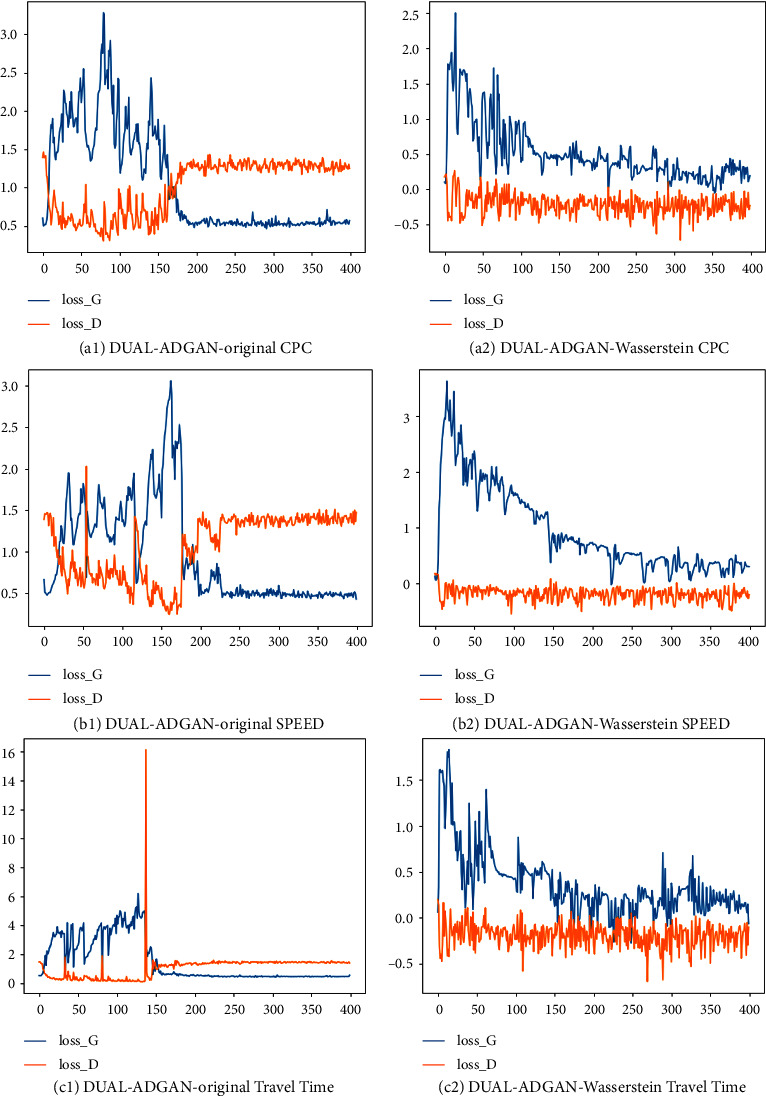
Training results of the DUAL-ADGAN model using two different loss functions on three datasets.

The training loss values of the DUAL-ADGAN anomaly detection model using two different loss functions on three datasets are shown in Figure 9, where DUAL-ADGAN-original indicates that the model uses the original generative adversarial network loss function and DUAL-ADGAN-Wasserstein indicates that the model uses the Wasserstein distance as the loss function. In the figure, (a1), (b1), and (c1) are the loss value plots on the three datasets during DUAL-ADGAN-original training, and (a2), (b2), and (c2) are the loss value plots on the three datasets during DUAL-ADGAN-Wasserstein training. The blue curve in the loss plot is the generator loss value, and the orange curve is the discriminator loss value. The discriminator and generator loss values of the DUAL-ADGAN-original model fluctuate greatly in the early training period on all three datasets, and the DUAL-ADGAN-original model needs an average of 160 rounds of training on the three datasets before it gradually converges. The DUAL-ADGAN-Wasserstein model starts a steady decline in loss values from the early stage of training. The reason for this is that the introduction of the Wasserstein distance allows the model to measure the distance between the two distributions well, even when there is no overlap between the generated sample distribution and the real sample distribution in the early training period. The experimental results demonstrate that using Wasserstein distance as the loss function of the generative adversarial network can stabilize the training of the model. For the anomaly detection problem, training stable and easily trainable generative adversarial networks is important to improve the performance of anomaly detection models.

### 4.3. Experiment Summary

Through the above experiments and the analysis of the experimental data, the experimental results obtained show the following:

1. The adoption of the Wasserstein distance satisfying the 1-Lipschitz constraint as the loss function of the generative adversarial network can effectively improve the stability of model training and enable the model to learn the distribution of real data stably and effectively.
2. Designing a time-series prediction task when reconstructing anomalies and increasing the differentiation between anomalous and normal data led to a significant improvement in the recall rate of anomaly detection, proving that the anomaly exposure mechanism can effectively alleviate the problem of anomaly miss detection in the anomaly detection model.
3. By introducing the loss function of Fence-GAN and training discriminators that are consistent with the anomaly detection target, the model effectively reduces the false detection rate of anomalous data when detecting anomalies.

## 5. Conclusions

In this paper, a generative adversarial network model DUAL-ADGAN for time-series anomaly detection is proposed, which jointly detects anomalous data in time series by reconstruction loss, prediction loss, and discriminative loss. The model learns the general distribution pattern of the temporal data in the dataset through the generator of WGAN and finds the inverse mapping of the test data in the random potential space, which is input to the generator of WGAN and reconstructs the sequence to obtain the reconstruction loss. To solve the problem that some of the exceptions are also well reconstructed, a data prediction module is added to the model to expose some of the reconstructed exceptions. In addition, the discriminator of Fence-GAN is used to replace the discriminator in the original GAN network to solve the problem that the original discriminator is not consistent with the anomaly detection target one. The experiments show that using Wasserstein distance as the loss function in the data reconstruction module improves model training stability and allows the model to converge faster during the actual training process. At the same time, the data reconstruction module in the model ensures the validity of the reconstructed data through WGAN and predictor module. The data discriminator module adopts the discriminator trained by the Fence-GAN loss function, which better solves the problem of inconsistency between the discriminator and the anomaly detection task and effectively alleviates the problem of missed and false detection in time-series anomaly detection. The results of anomalous data detection on three real datasets show that the DUAL-ADGAN model is more effective, reliable, and accurate in detecting anomalous data in time-series data.

In further research, the focus will be on how to reduce the presence of false detection rates during anomaly detection. For a specific dataset, anomaly filtering rules can be formulated to better match the characteristics of the dataset, and the anomaly data detected by the anomaly detection model can be further filtered according to the anomaly filtering rules to reduce the false detection rate of the anomaly detection model, making the anomaly detection model better able to complete the anomaly detection task in complex environments.

## Figures and Tables

**Algorithm 1:**
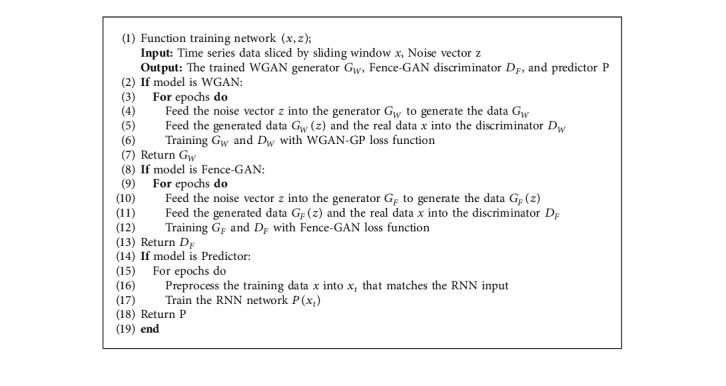
DUAL-ADGAN training network model pseudocode.

**Algorithm 2:**
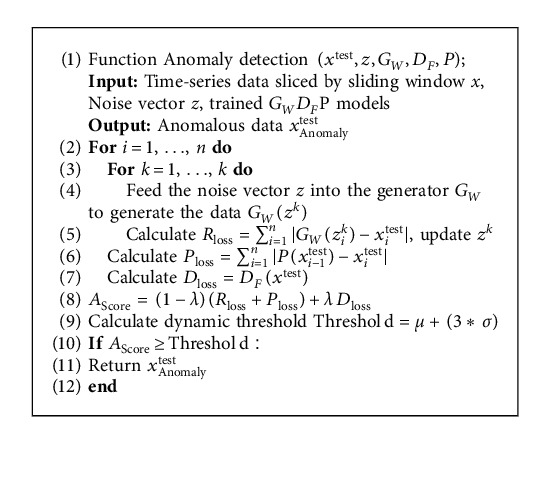
DUAL-ADGAN anomaly detection network model pseudo-code

**Table 1:** Network structure parameters.

Parameters	WGAN generator	WGAN discriminator	Fence-GAN generator	Fence-GAN discriminator	Predictor
Layers	3^∗^LSTM	1^∗^LSTM	3^∗^LSTM	1^∗^LSTM	1^∗^RNN
Hidden cells	100	100	100	100	50
Activation	Tanh	None	Tanh	Sigmoid	ReLU

**Table 2:** Comparison of the performance of each anomaly detection model.

Model	RealAdExchange-CPC				RealTraffic-SPEED				RealTraffic-TravelTime				Avg
	Acc	Pre	Recall	F1	Acc	Pre	Recall	F1	Acc	Pre	Recall	F1	F1
K-means [17]	0.737	0.407	0.73	0.523	0.857	0.474	0.6	0.529	0.863	0.64	0.666	0.653	0.568
OC-SVM [20]	0.876	0.571	0.834	0.678	0.892	0.588	0.667	0.625	0.887	0.611	0.785	0.687	0.663
LOF [16]	0.884	0.625	0.769	0.689	0.911	0.692	0.6	0.642	0.9	0.653	0.8	0.719	0.683
IF [19]	0.908	0.785	0.733	0.758	0.937	0.666	0.8	0.727	0.939	0.744	0.842	0.790	0.758
LSTM-AE [22]	0.906	0.75	0.774	0.761	0.938	0.785	0.734	0.758	0.883	0.633	0.679	0.655	0.725
NSIBF [24]	0.96	0.92	0.851	0.885	0.927	0.88	0.815	0.846	0.96	0.892	0.846	0.868	0.866
Mad-GAN [29]	0.915	0.72	0.75	0.735	0.946	0.846	0.734	0.786	0.932	0.783	0.763	0.773	0.765
Fence-GAN [34]	0.914	0.71	0.73	0.72	0.929	0.7857	0.688	0.734	0.94	0.848	0.737	0.789	0.748
Tad-GAN [30]	0.942	0.8	0.889	0.842	0.938	0.789	0.834	0.81	0.938	0.828	0.763	0.795	0.816
DUAL-ADGAN	0.962	0.848	0.967	0.903	0.949	0.818	0.9	0.857	0.96	0.845	0.926	0.883	0.881

**Table 3:** Ablation experiments results.

Dataset	Model	Acc	Pre	Recall	F1
RealAdExchange-CPC	Wad-GAN	0.941	0.857	0.75	0.8
	WganG_FenceD	0.948	0.8	0.923	0.857
	DUAL-ADGAN	0.962	0.848	0.967	0.903

RealTraffic-SPEED	Wad-GAN	0.912	0.734	0.647	0.688
	WganG_FenceD	0.921	0.786	0.647	0.71
	DUAL-ADGAN	0.949	0.818	0.9	0.857

RealTraffic-TravelTime	Wad-GAN	0.947	0.823	0.8	0.812
	WganG_FenceD	0.956	0.846	0.868	0.857
	DUAL-ADGAN	0.96	0.845	0.926	0.883

## Data Availability

The data used to support the findings of the study are included in the article at https://www.kaggle.com/datasets/boltzmannbrain/nab.
